# Supplementary material for: Mapping genetic variants for nonsense-mediated mRNA decay regulation across human tissues
Source: Genome Biol. 2023 Jul 11;24:164. doi: 10.1186/s13059-023-03004-w (PMC10337212; doi:10.1186/s13059-023-03004-w)
Supplement: Supplementary file 1 — Additional file 1. Supplementary Texts and Supplementary Figures S1-S8. [file 13059_2023_3004_MOESM1_ESM.pdf]

Supplementary Texts

Expression normalization. We performed NMD-QTL mapping for 48 tissues from GTEx with the sample size ≥ 70. For a given tissue, the NMD expression and non-NMD expression were obtained by the following procedures:

- Lowly expressed genes were filtered out. Only genes with TPM > 0.1 in at least 10 samples were selected for further analysis.
- The filtered genes expression was summarized into NMD expression and non-NMD expression.
- Since NMD expression could be sparse, only genes with the percent of zeros less than 40% in NMD expression were kept.
- Phenotypic traits (NMD expression and non-NMD expression) were normalized based on a normalization factor. Suppose the total number of genes is G and the total number of individuals is K, the normalization factor for each sample k was calculated based on all expressed genes as follows:
    - First a geometric mean was calculated for each gene g across samples:

$$\mu^{(g)} = \left(\prod_{k:TPM_g^{(k)} \neq 0} TPM_g^{(k)} \right)^{1/\sum_k I\left(TPM_g^{(k)} \neq 0\right)}$$

    - Then, the normalization factor for each individual k is obtained as the median fold-change of expressed genes compared to this geometric mean: $\delta^{(k)} = \mathrm{median}\left(\frac{\mathrm{TPM}_g^{(k)}}{\mu^{(g)}},\ \mathrm{for}\ g \in G\right)$
    - then the NMD expression and non-NMD expression were normalized by this normalization factor, more formally, normalized TPM for every gene g and sample k is then given by: $TPM'^{(k)}_g = \frac{TPM_g^{(k)}}{\delta^{(k)}}$.

- Finally, NMD expression and non-NMD expression were further normalized across samples by the rank-based inverse normal transform used in FastQTL for NMD-QTL mapping.

Python code snippet for the normalization logic and the inverse normal transform is shown below.

```
import pandas as pd
import scipy.stats as ss
from scipy.stats.mstats import gmean
def rank_INT(x, c=3./8):
    """Rank-based Inverse Normal Transform.
     Ties share the same value after transformation."""
    n = len(x)
    r = ss.rankdata(x, method='average')
    return ss.norm.ppf((r - c) / (n - 2 * c + 1))

# normalization factors
gene_tpm = pd.read_csv(os.path.join(base_path, 'GTEx_Analysis_2016-01-
15_v7_RNASeQCv1.1.8_gene_tpm.gct.gz'),
            skiprows=2, sep='\t', usecols=tissue_cols[2:])
gene_tpm = gene_tpm[(gene_tpm > .1).sum(axis=1) >= 10]  # filter out lowly expressed genes
genes_gmean = gene_tpm.apply(lambda row: gmean(row[row != 0]), axis=1)
norm_factors = gene_tpm.divide(genes_gmean, axis=0).median(axis=0)
```

Detailed parameter settings used in simulations. Without any prior knowledge on how the NMD effect should be, parameters $\Theta(t , \alpha , \theta)$ were generated by first drawing α_A and θ_A from a uniform distribution U(0.2, 0.8), and then obtaining α_a, and θ_a by ensuring the effect size $|\alpha_A - \alpha_a|$ and $|\theta_A - \theta_a|$ belonging to the desired range. For cases assuming $t_A=t_a=t$ we chose t = 4, and for cases assuming $t_A \neq t_a$, we drew both $t_A \sim U(3,5)$ and $t_a \sim U(3,5)$, and then applied a Gaussian noise term. We drew the Gaussian noise term $\sigma \sim N(0,1.5)$ and truncated y_i to be non-negative to mimic the real data.

Hyper-geometric test used in disease SNP colocalization study for NMD-QTLs. For a given disease d_i, let N be the total number of NMD-QTLs discovered, M be the universal set of SNPs (here we assume it is the number of all SNPs tested in GTEx), let n be the number of markers reported in DisGeNET for d_i, and x be the number of NMD-QTLs that were indeed the disease marker for d_i. Then the p-value for the test is given by

$$p = \mathbf{Pr}(X \geq x - 1)$$

where X follows a hypergeometric distribution of which the probability mass function is defined as

$$p(x, M, n, N) = \frac{\binom{n}{x}\binom{M-n}{N-x}}{\binom{M}{N}}.$$

Proportion test used in disease SNP colocalization study for NMD-QTLs. For a given disease d_i, four statistics were gathered as follows. The alternative hypothesis was $H_A: \frac{x_1}{y_1} > \frac{x_2}{y_2}$.

- x_1: number of NMD-QTLs reported to be associated with the d_i
- y_1: total number of NMD-QTLs reported to be associated diseases
- x_2: number of eQTLs reported to be associated with the d_i
- y_2: total number of eQTLs reported to be associated diseases

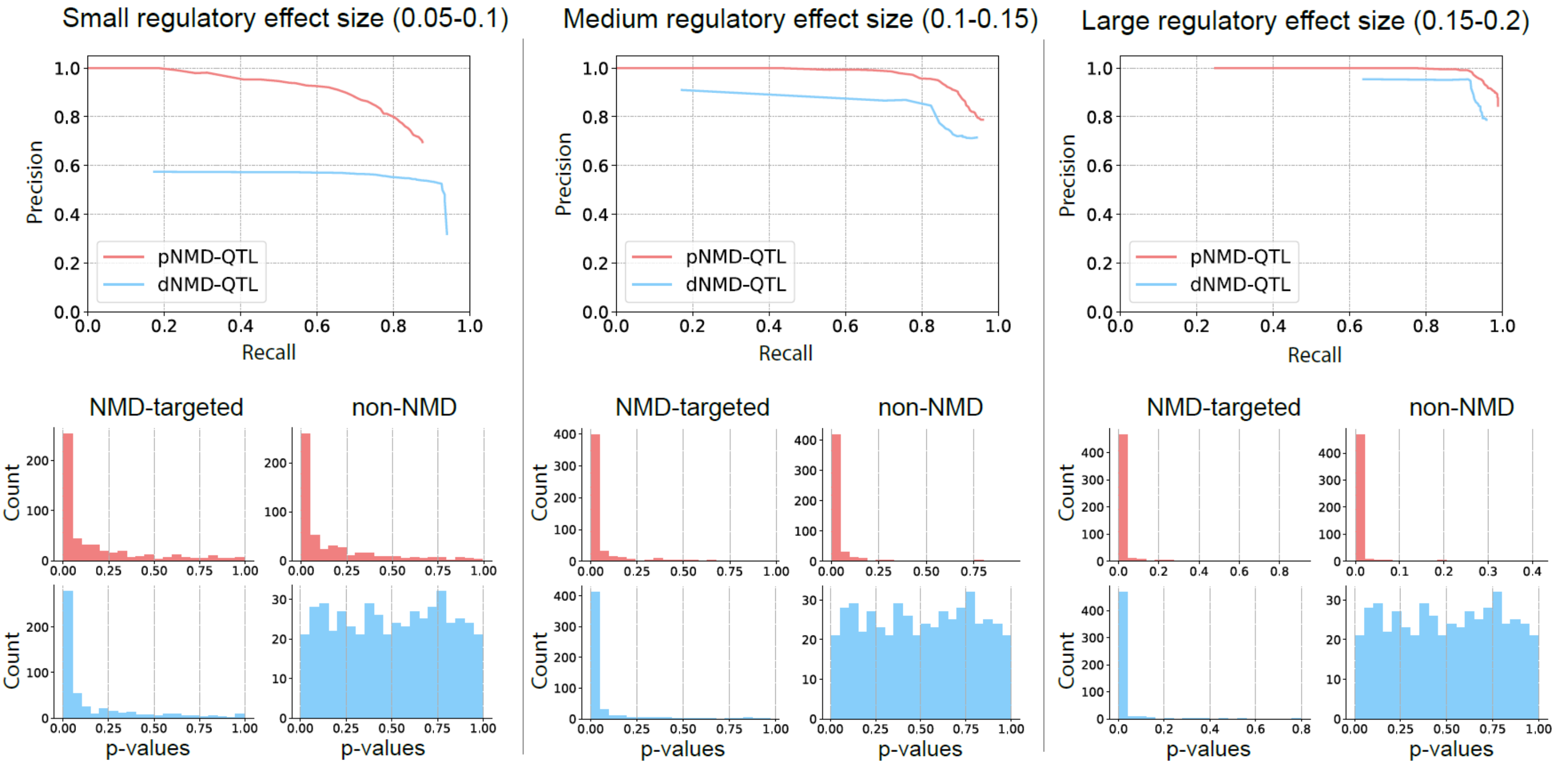

Figure S1. NMD-QTL identification results for simulations with various effect sizes. The top panels show the Precision-Recall curves for small effect size (0.05-0.1), medium effect size (0.1-0.15), and large effect size (0.15-0.2). The bottom panels show the p-value distributions for the allelic effect for NMD-targeted transcripts and non-NMD transcripts.

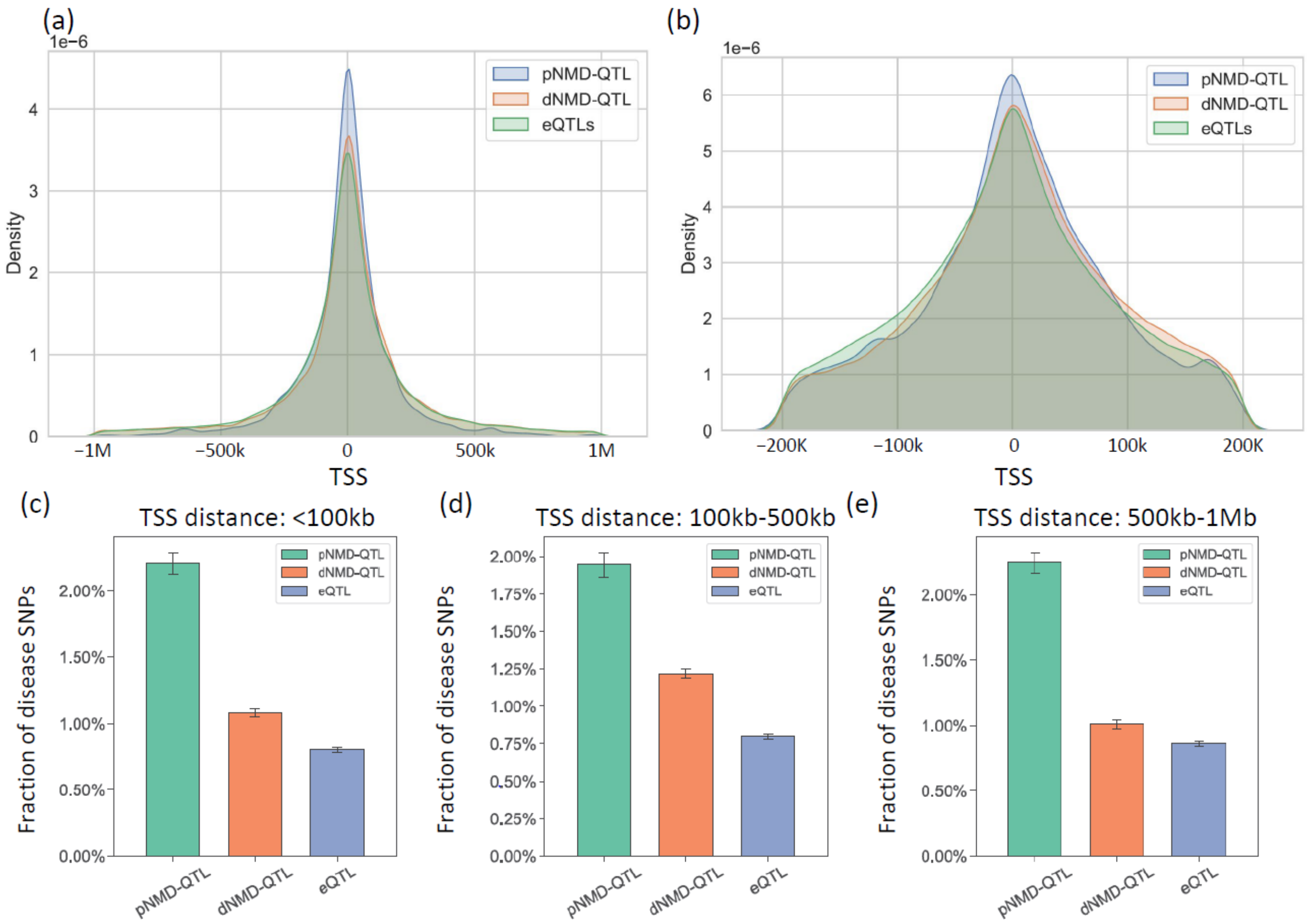

Figure S2. Location preference of NMD-QTLs and their overlap with disease SNPs. (a) Distribution of the distance to the transcription start site (TSS) for NMD-QTLs and eQTLs within 1 Mb regions of the TSS. (b) Distribution of the distance to the TSS for NMD-QTLs and eQTLs within 200 kb regions of the TSS. (c) Overlapping of QTLs within 100 kb of the TSS with disease SNPs. (d) Overlapping of QTLs within 100 kb to 500 kb of the TSS with disease SNPs. (e) Overlapping of QTLs within 500 kb to 1 Mb of the TSS with disease SNPs.

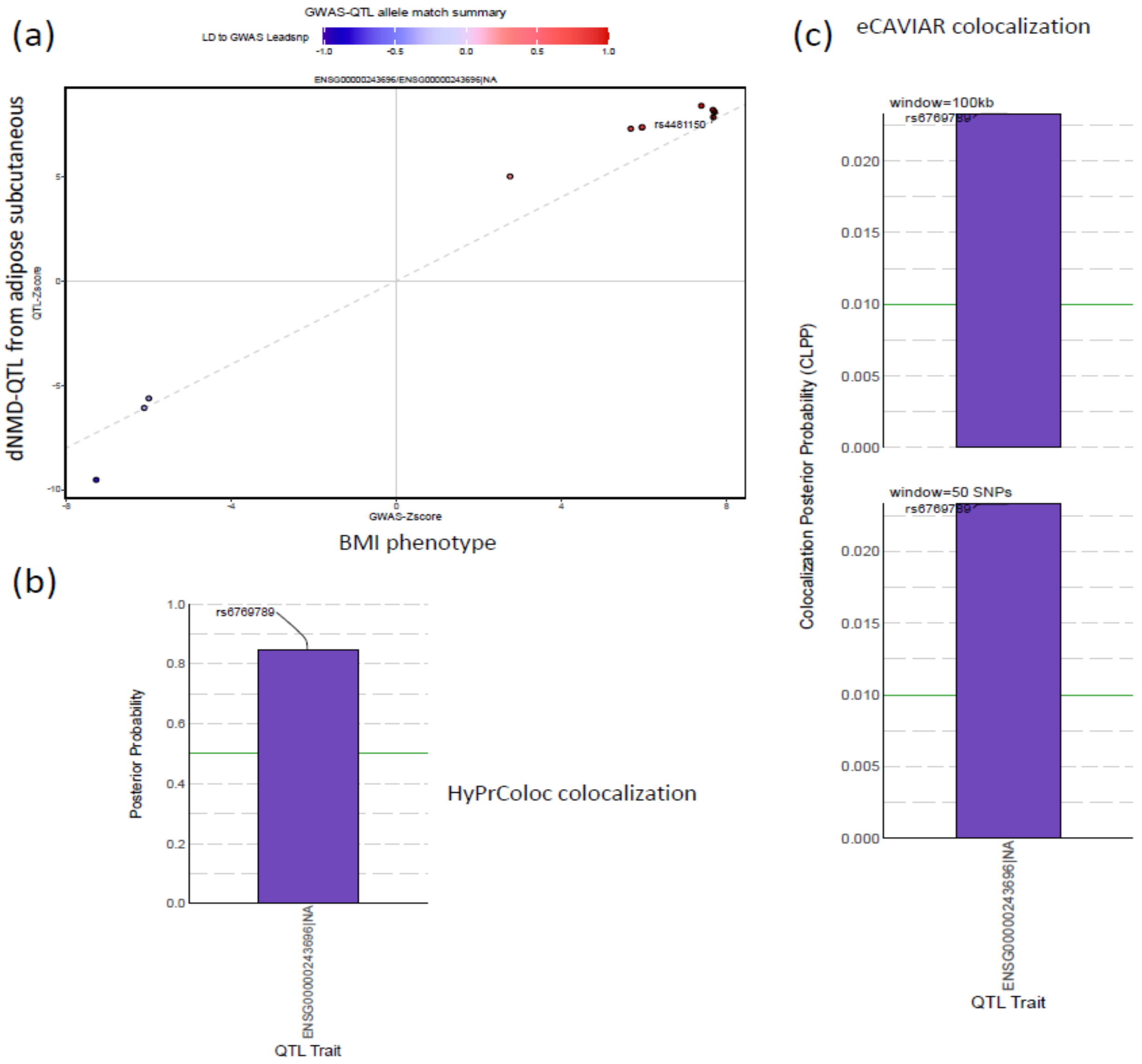

Figure S3. Colocalization analysis between dNMD-QTLs and body mass index (BMI) for gene ENSG00000243696. The dNMD-QTLs were derived from adipose (subcutaneous) tissue. SNP rs6769789 is highlighted as a potential causal NMD-QTL for BMI. **(a)** Correlation of Z-scores between NMD-QTLs and GWAS signals. **(b)** HyPrColoc colocalization result for rs6769789. **(c)** eCAVIAR colocalization result for rs6769789.

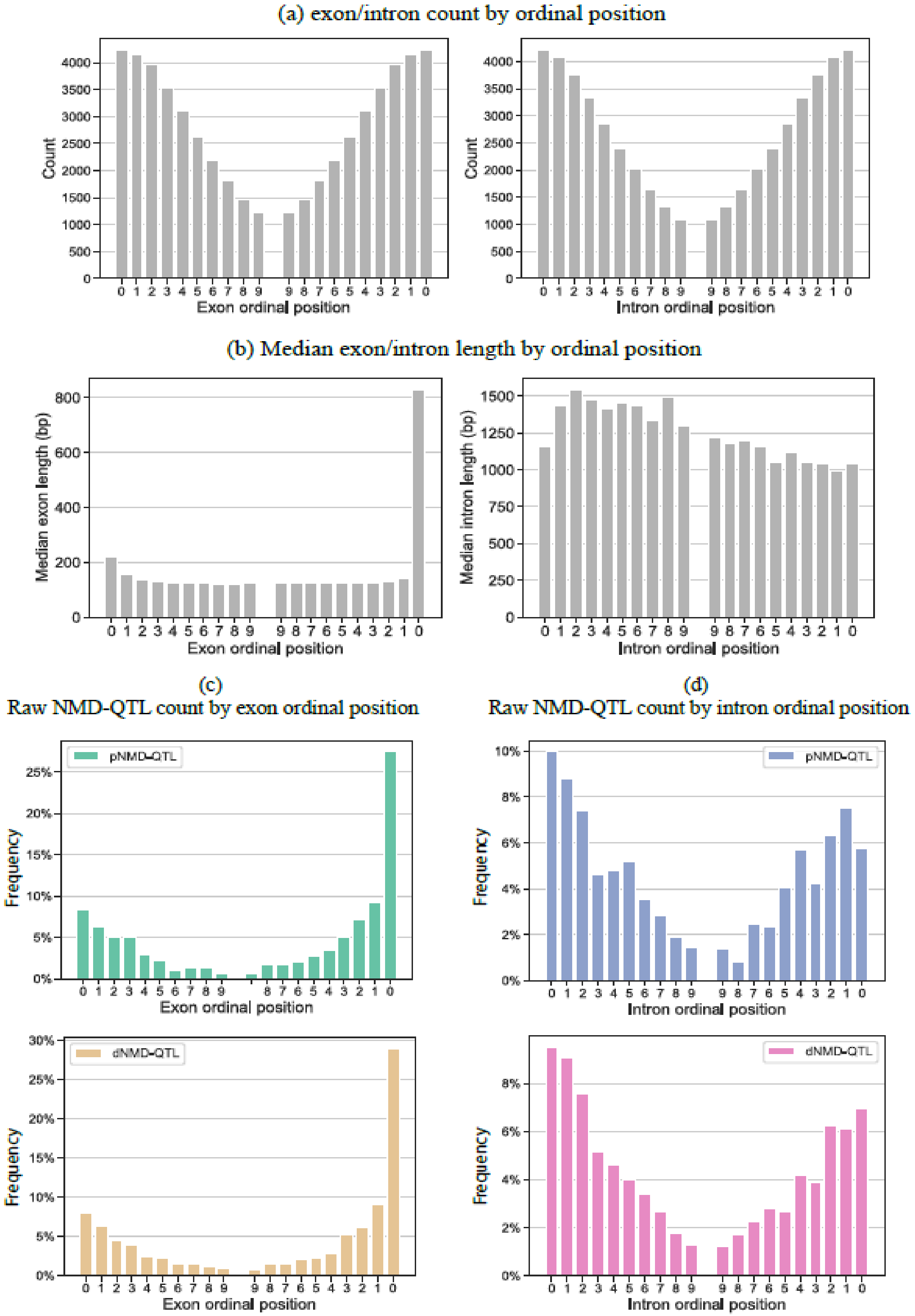

Figure S4. Ordinal positions of exon and intron intervals in NMD genes. **(a)** The exon or intron interval count per ordinal position. **(b)** The median exon or intron length per ordinal position. **(c)** Distribution of the raw pNMD-QTL or dNMD-QTL count per exon ordinal position. **(d)** Distribution of the raw pNMD-QTL or dNMD-QTL count per intron ordinal position.

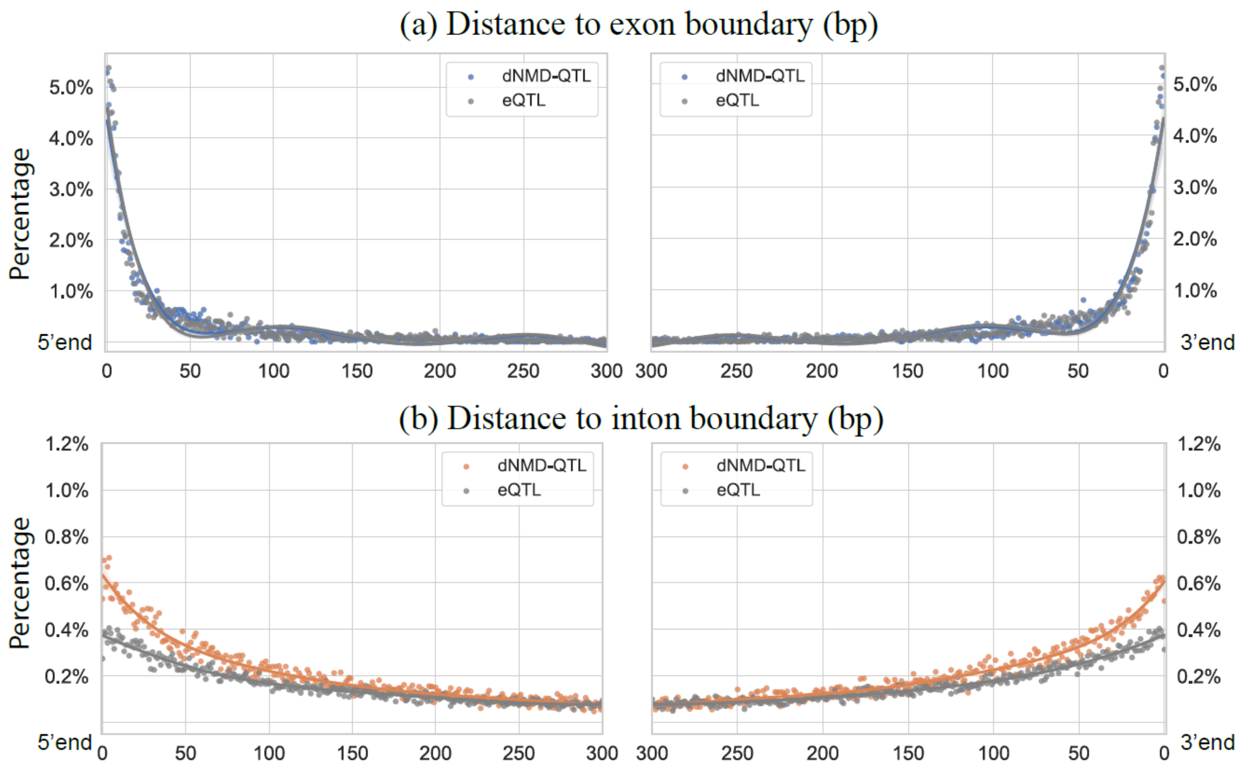

Figure S5. Distances to exon (or intron) boundaries for dNMD-QTLs or eQTLs located on an exon (or intron).

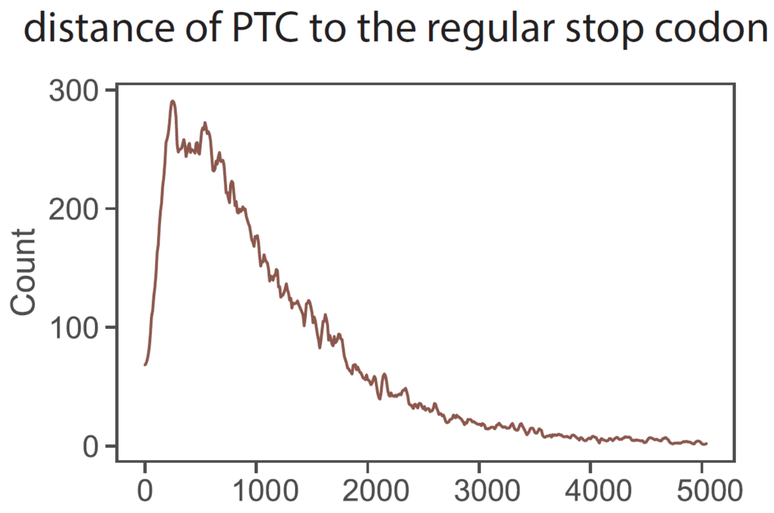

Figure S6. The distance between the PTC of the NMD-targeted transcripts and the regular stop codon located on the last exon of the non-NMD transcripts. Only exonic positions are counted in the distance calculation.

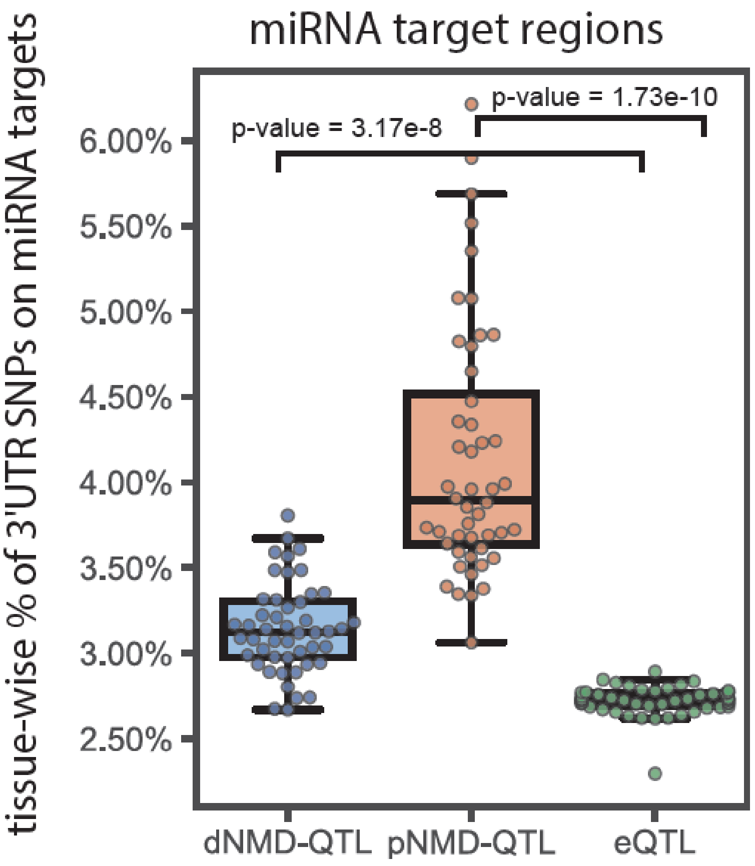

Figure S7. Tissue-wise percentages of 3' UTR NMD-QTLs in miRNA targets compared to those of 3' UTR eQTLs. The Y axis shows the percentage of NMD-QTLs locating in miRNA targets among all NMD-QTLs in 3' UTRs identified from a specific tissue.

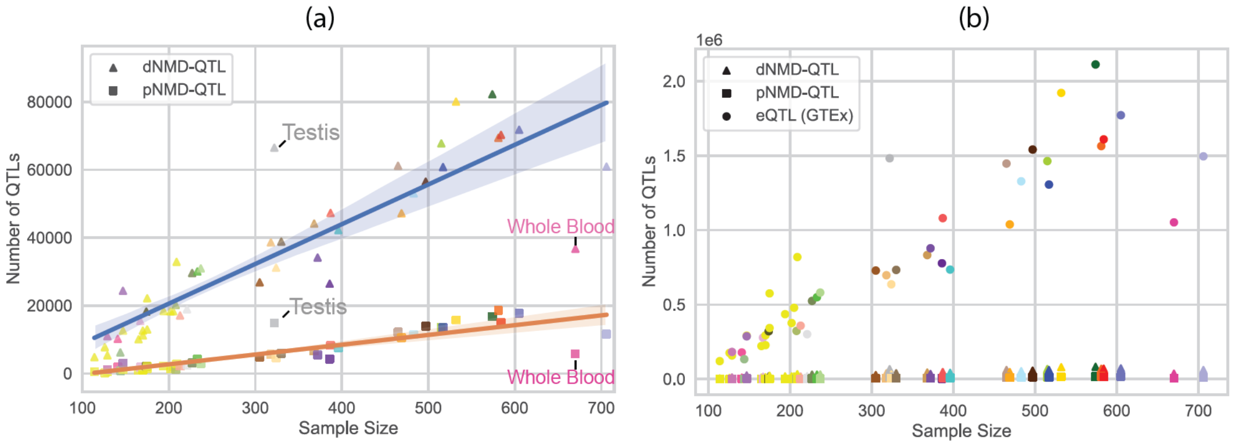

Figure S8. Detection power and sample size. **(a)** Positive correlation between the number of NMD-QTLs identified for each tissue and the tissue sample size (i.e., the number of donors). **(b)** The positive correlation observed for GTEx eQTLs as well.
