# Supplementary material for: Mapping genetic variants for nonsense-mediated mRNA decay regulation across human tissues
Source: Genome Biol. 2023 Jul 11;24:164. doi: 10.1186/s13059-023-03004-w (PMC10337212; doi:10.1186/s13059-023-03004-w)
Supplement: Supplementary file 6 — Additional file 6. Review history. [file 13059_2023_3004_MOESM6_ESM.docx]

**1^st^ round**

**Reviewer 1**

In this manuscript, Sun and Chen attempt to explore the impact of genetic variants on gene expression regulation through Nonsense-mediated mRNA decay. The draft describes interesting observations that would benefit from further support from other sources based on experimental validation focusing exclusively on NMD-related degradation events. On the downside, the manuscript does not bring novel insight into the interplay between these variants and NMD, and it is often limited to descriptive observations based on statistical correlations. Therefore, eventhough the work could be of interest to individuals in the fielad it is not of outstanding interest to a broad audience.

I am providing here a detailed list of major and minor points to be addressed:

Major comments:
1. Overall, in several points of the manuscript, it is not clear whether the analysis is gene- or isoform-specific. For instance, on p. 17, lines 14-45, the analysis is described as transcript-specific, and it is elusive how different isoforms of the same gene were told apart. So far, an NMD isoform-specific analysis has only been performed by combining short, and long-read sequencing reads. Along these lines, it is crucial to consider which ORF is regarded as the ORF of the principal isoform to define precisely where premature termination codons are introduced.
2. It is unclear what exact specifications are considered to characterize an allele as NMD-sensitive. As written on p. 6, l. 10-14, the annotation of GENCODE seems to be the unique parameter considered. It would be preferable to apply more filtering steps, such as the relative position of the PTC compared to the exon junction positions of the corresponding transcripts, or limit the analysis to genes that have been experimentally validated to give rise to translated transcripts (for instance via ribosome profiling).
3. It is essential to validate whether the applied pipeline can detect well-known NMD targets revealed from NMD-Sequencing experiments. An isoform-specific analysis has been performed by Karousis et al. 2021.
4. Concerning the RNA binding proteins detected, were hnRNPL1 (Kishor et al., 2018 DOI: 10.15252/embj.201899128) or PTBP1 (Ge et al., 2016 doi: 10.7554/eLife.11155) de-riched on NMD-QTLs? These two proteins are expected to protect mRNAs from degradation, and abrogation of binding renders the corresponding transcripts vulnerable to degradation.

Minor comments
1. It has been known for more than a decade that NMD also targets endogenous mRNAs; therefore, the term «recent» (p.1, line 51, p. 3, line 54) may be redundant.
2. P.7, line 7: please define allelic effect size and how this is perceived for an NMD-related mutation.
3. Discuss the general connection between NMD and the brain. This connection is apparent, for instance, through the impact of NMD factors mutations in neuronic development and diseases.
4. Minor typo on p. 1, Line 50: delete "s" from expressions
5. P.6, line 47: To improve clarity, consider replacing the terms "αs or θs" with the phrase "α or θ values".

**Reviewer 2**

In this study, Sun and Chen introduce the concept of NMD-QTLs, and perform a systematic analysis, based on GTEx data, for SNPs that are correlated with NMD. Testing genes with transcripts that are assumed to be subjected to NMD regulation (by Gencode annotations) - 'NMD genes' - the authors define two types of NMD-QTLs: pNMD-QTLs associated with the proportion of NMD transcripts generated from an NMD gene, and dNMD-QTLs that are associated with degradation efficiency by NMD. Analyzing 48 GTEx tissues, a total of >54k pNMD-QTLs and ~320k dNMD-QTLs were detected. These putative NMD-associated SNPs were then further characterized in terms of tissue specificity, overlap with disease-associated variants, location, possible mechanism of action (i.e., overlap with miR and RBP binding sites) and overlap with splicing-QTLs.
The concept of NMD-QTL is novel and the NMD-associated SNPs identified by this study can potentially shed light on NMD mechanisms of action, and, importantly, on its biological impact and links to human traits and diseases. I have some concerns on some of the statistical analyses that could be improved to sharpen the discoveries.

1. A main issue is the 'colocalization' analyses performed against GWAS signals (disease-associated SNPs). The authors intersected NMD-QTLs and GWAS SNPs to find overlaps. However, given the overabundance of GWAS and eQTL SNPs, many overlaps are expected by chance. That is, many of the overlaps detected in this study are probably spurious. (This issue is emphasized by this ref: PMID: 31043754). Several statistical methods were developed to cope with this challenge (e.g., ColocQuiaL, eCAVIAR) and the authors should use such methods to distinguish overlaps that indicate shared mechanism from spurious ones. In addition, in joint analysis of QTLs and GWAS SNPs, the 'colocalization' is typically used for such tests, and therefore the use of this term by the authors is confusing. For the intersection analyses as done throughout this study, it would be better to use 'overlap' (or a similar term) rather than 'colocalization'.

2. I am concerned about rate of false calls of NMD QTLs. dNMD-QTLs are called when the association is only observable for NMD-targeted transcripts but not for non-NMD transcripts.

a. It is not clear what 'observable' means here? q-value < 0.05?
b. If so, then if q-value<0.05 is observed for NMD-Txs and q-value>=0.05 is observed for the non-NMD Txs, a dNMD-QTL is called? If this is the case, it is very possible to get such effect just due to difference in the power of the tests of the NMD and non-NMD Txs. To get some estimate of false calls, the authors could, as a control, repeat the same analysis on the non-NMD genes; split each such gene to two random sets of transcripts (in a way that simulates the division of transcripts of NMD genes to NMD and non-NMD transcripts), and compare the prevalence of "NMD" QTLs detected by such control to the one obtained on the NMD genes.

3. The analysis on the overlap between splicing-QTLs and NMD-QTLs seems circular to me: the two tests are interlinked and are actually based on examination of the highly coupled features. This is exemplified by the NMD-genes with direct evidence for AS-NMD coupling. Consider such case, in which the gene has only one NMD transcript. The test for association with the level of that transcript is highly coupled with the test for association of the exon that defines that transcript (the exon containing the PTC). So statistically, these two highly related tests are bound to yield highly overlapping results.

4. As for the enrichment of NMD-QTL for miR BSs compared to eQTLs:
a. As TragetScan miR BS are located in 3'UTRs, only 3'UTR SNPs should be included. If this is done, do NMD-QTLs still show enrichment for miR BSs compared to eQTL?
b. Figure 9: the label of the Y axis in panels a, c and e says 'enrichment'. But the Y axis doesn't show enrichment. In addition, a global test for enrichment was done, but not per tissue. Are all tissues show significant enrichment?

5. For NMD-QTLs that were detected in many tissues (say, >20), could the authors examine and characterize the consistency of allele effect across the tissues?

6. In many places, the claims made by the authors should be toned down. For example, p11 line 43 "suggesting that NMD regulation is an essential component"; p12 line 48: ' .. is a key component of NMD regulation …'; p13 20: 'interplay closely with DNA repair and is essential in ensuring genome integrity'. Such strong claims should be based on some quantification of importance (or experimental validation). In the lack of such supporting evidence, the claims should be much weakened.

7. One limitation of the analysis performed in this study is the consideration of all gene's transcripts annotated as NMD-transcripts collectively. For an NMD-gene with multiple NMD-Txs, it is possible that an NMD-SNP would affect only one of the NMD-Txs, and therefore, its effect would be diluted when considering all NMD-TXs together. This limitation could be discussed in the Discussion.

**Authors’ response**

Reviewer 1

[Summary]

In this manuscript, Sun and Chen attempt to explore the impact of genetic variants on gene expression regulation through Nonsense-mediated mRNA decay. The draft describes interesting observations that would benefit from further support from other sources based on experimental validation focusing exclusively on NMD-related degradation events. On the downside, the manuscript does not bring novel insight into the interplay between these variants and NMD, and it is often limited to descriptive observations based on statistical correlations. Therefore, even though the work could be of interest to individuals in the field it is not of outstanding interest to a broad audience.

Responses:

First, we greatly appreciate your time and effort in reviewing our manuscript. Our primary goal of this work is to identify genetic variants associated with NMD regulation. We also described the properties of NMD-QTLs that we observed through integrative analysis with other data: (1) Enrichment of disease SNPs for NMD-QTLs; (2) Tissue-specificity of NMD-QTLs; (3) Specific genomic location preferences for NMD-QTLs; (4) Enrichment of RBP binding sites and miRNA binding sites for NMD-QLTs; (5) Enrichment of sQTLs for NMD-QTLs.

While we acknowledge that the detailed mechanism underlying these variants and NMD regulation is an essential step, we believe that identifying NMD-QTLs itself is of great interest to a broad audience. As we mentioned in the manuscript, variations in NMD efficiency are observed across individuals. Such variations can influence the outcome of PTC read-through therapeutic strategies aimed at suppressing nonsense mutations to restore full-length proteins, as the efficacy of such nonsense suppression largely depends on NMD efficiency. Our identified NMD-QTLs can help us to understand why some individuals are less responsive to the PTC read-through treatment. Therefore, identifying NMD-QTLs can have significant implications for personalized medicine without further mechanism analysis.

In conclusion, our study identifies genetic variants related to NMD efficiency and contributes to understanding NMD regulation. Our findings will be of interest to the scientific community and contribute to the development of personalized medicine strategies.

[Major comments]

1. Overall, in several points of the manuscript, it is not clear whether the analysis is gene- or isoform-specific. For instance, on p. 17, lines 14-45, the analysis is described as transcript- specific, and it is elusive how different isoforms of the same gene were told apart. So far, an NMD isoform-specific analysis has only been performed by combining short, and long-read sequencing reads. Along these lines, it is crucial to consider which ORF is regarded as the ORF of the principal isoform to define precisely where premature termination codons are introduced.

Responses:

Thanks for the question. We now modified the manuscript to make it clearer.

First, we performed genome-wide NMD-QTLs mapping in a gene-level fashion. But different from a typical gene-level analysis where all isoforms of a gene are considered together, we performed a specific grouping for NMD-targeted and non-NMD transcripts. Here we define NMD-targeted transcripts based on the transcript-level annotation in GENCODE; if the coding sequence of a transcript finishes >50bp from a downstream splice site, then it is tagged as an NMD-targeted transcript. Only genes with both NMD-targeted and non-NMD transcripts were considered and referred to as NMD-genes. For each NMD-gene, we have two expression quantifications: one summarized from NMD-targeted transcripts and another from non-NMD transcripts. We run QTL mapping for both expression quantifications separately. The pNMD- QTLs and dNMD-QTLs are then identified by the different characteristics of the effect sizes for these two expression phenotypes.

Secondly, to study the position preferences of NMD-QTLs along exons and introns (Figs. 7, 8a, and 8b), we used what was referred to as the “collapsed gene model” for exon/intron intervals and gene boundary locations. This procedure collapses all isoforms from a gene into a “single- transcript gene” by unionizing all exon intervals and several other considerations. This has been widely used in the GTEx gene modeling, and a detailed explanation can be found on the GTEx FAQ page (https://www.gtexportal.org/home/faq).

Finally, when we consider the distance between NMD-QTLs and premature termination codons (PTCs) or regular stop codons in Fig. 8c (the mentioned place by reviewer 1: p17 line14-45) and Fig. 8d, we did it on the transcript level. The distance between an NMD-QTL and each transcript isoform's PTC/regular stop codon was calculated separately. All these distances are pooled to be presented in the figures.

2. It is unclear what exact specifications are considered to characterize an allele as NMD- sensitive. As written on p. 6, l. 10-14, the annotation of GENCODE seems to be the unique parameter considered. It would be preferable to apply more filtering steps, such as the relative position of the PTC compared to the exon junction positions of the corresponding transcripts, or limit the analysis to genes that have been experimentally validated to give rise to translated transcripts (for instance via ribosome profiling).

Responses:

We are sorry for the confusion. The filtering steps suggested here have already been considered when GENCODE annotates NMD transcripts. A transcript will be tagged as NMD if

(i) there is a PTC located >50bp from a downstream splice site; or

(ii) if the transcript does not cover the full reference coding sequence then it is annotated as NMD if NMD is unavoidable (i.e., no matter what the exon structure of the missing portion is, the transcript will be subject to NMD).

We have added the details in the revisions (1st paragraph, page 28).

3. It is essential to validate whether the applied pipeline can detect well-known NMD targets revealed from NMD-Sequencing experiments. An isoform-specific analysis has been performed by Karousis et al. 2021.

Responses:

As we mentioned, our analysis methods are to identify genetic variants associated with NMD- genes. Those NMD-genes are based on GENCODE annotation, including well-known NMD targets such as GADD45B, HNRNPL, SRSF10, and BAG1 which was studied in Karousis et al. 2021. We discovered 11 dNMD-QTLs for BAG1, ten dNMD-QTLs and one pNMD-QTL for HNRNPL, seven dNMD-QTLs for GADD45B, and three dNMD-QTLs for SRSF10. These have been added on page 9.

4. Concerning the RNA binding proteins detected, were hnRNPL1 (Kishor et al., 2018 DOI: 10.15252/embj.201899128) or PTBP1 (Ge et al., 2016 doi: 10.7554/eLife.11155) de-riched on NMD-QTLs? These two proteins are expected to protect mRNAs from degradation, and abrogation of binding renders the corresponding transcripts vulnerable to degradation.

Responses:

Thanks for the insight about these two specific RBPs. Upon checking, the binding sites of the two RBPs are indeed de-enriched on NMD-QTLs. For example, in the K562 cell line, the total length of PTBP1 binding sites accounted for 0.6% of all RBP binding positions, but only 0.21% of dNMD-QTLs and 0.16% of pNMD-QTLs residing in RBP binding sites were on PTBP1 binding sites (p-values for the de-enrichment < 2e−10). Similarly, the total length of HNRNPL binding sites accounted for 2.5% of all RBP binding positions, but only 1% dNMD-QTLs and 1% pNMD-QTLs residing in RBP binding sites were on HNRNPL binding sites (p-values < 2e−16). Similarly, in the HepG2 cell line, the total length of PTBP1 binding sites accounted for 1.4% of the total RBP binding site positions, but only 0.27% of dNMD-QTLs and 0.34% of pNMD-QTLs residing in RBP binding sites were on PTBP1 binding sites (p-values < 2e−16). The total length of HNRNPL binding sites accounted for 2.6% of the total RBP binding positions, but only 1.1% of dNMD-QTLs and 0.9% pNMD-QTLs residing in RBP binding sites were on HNRNPL binding sites (p-values < 2e−16). These have been added on page 20.

[Minor comments ]

1. It has been known for more than a decade that NMD also targets endogenous mRNAs; therefore, the term «recent» (p.1, line 51, p. 3, line 54) may be redundant.

Responses: We revised the sentences and removed “recent.”

2. P.7, line 7: please define allelic effect size and how this is perceived for an NMD-related mutation.

Responses: We now added the definition on page 7. Allelic effect size here is the magnitude of the effect that an allele has on the expression level of NMD-targeted transcripts or non-NMD transcripts. Mathematically, it is the linear regression slope in our NMD-QTL modeling.

3. Discuss the general connection between NMD and the brain. This connection is apparent, for instance, through the impact of NMD factors mutations in neuronic development and diseases.

Responses: We now added more discussion about this connection on page 24.

4. Minor typo on p. 1, Line 50: delete "s" from expressions

Responses: Thanks for pointing it out. We deleted the “s.”

5. P.6, line 47: To improve clarity, consider replacing the terms "αs or θs" with the phrase "α or θ values".

Responses: Corrections have been made.

Reviewer 2

[Summary]

In this study, Sun and Chen introduce the concept of NMD-QTLs, and perform a systematic analysis, based on GTEx data, for SNPs that are correlated with NMD. Testing genes with transcripts that are assumed to be subjected to NMD regulation (by Gencode annotations) - 'NMD genes' - the authors define two types of NMD-QTLs: pNMD-QTLs associated with the proportion of NMD transcripts generated from an NMD gene, and dNMD-QTLs that are associated with degradation efficiency by NMD. Analyzing 48 GTEx tissues, a total of >54k pNMD-QTLs and ~320k dNMD-QTLs were detected. These putative NMD-associated SNPs were then further characterized in terms of tissue specificity, overlap with disease-associated variants, location, possible mechanism of action (i.e., overlap with miR and RBP binding sites) and overlap with splicing-QTLs.

The concept of NMD-QTL is novel and the NMD-associated SNPs identified by this study can potentially shed light on NMD mechanisms of action, and, importantly, on its biological impact and links to human traits and diseases. I have some concerns on some of the statistical analyses that could be improved to sharpen the discoveries.

Responses:

We greatly appreciate your time and effort in reviewing our manuscript. This is a good summary of our work. We kindly addressed your comments below.

[Comments]

1. A main issue is the 'colocalization' analyses performed against GWAS signals (disease- associated SNPs). The authors intersected NMD-QTLs and GWAS SNPs to find overlaps. However, given the overabundance of GWAS and eQTL SNPs, many overlaps are expected by chance. That is, many of the overlaps detected in this study are probably spurious. (This issue is emphasized by this ref: PMID: 31043754). Several statistical methods were developed to cope with this challenge (e.g., ColocQuiaL, eCAVIAR) and the authors should use such methods to distinguish overlaps that indicate shared mechanism from spurious ones. In addition, in joint analysis of QTLs and GWAS SNPs, the 'colocalization' is typically used for such tests, and therefore the use of this term by the authors is confusing. For the intersection analyses as done throughout this study, it would be better to use 'overlap' (or a similar term) rather than 'colocalization'.

Responses:

Thanks for suggesting it. We now correct and use ‘overlap’ rather than ‘colocalization’ in the revised manuscript. Since our disease SNPs are those reported SNPs from many GWAS (disease SNPs assembled in the DisGeNET database) instead of a single GWAS, we cannot simply apply colocalization methods. ColocQuiaL and eCAVIAR are designed to relate signals from a GWAS to eQTLs/sQTLs identified in GTEx. Here, we are interested in the relationship between NMD-QTLs and disease SNPs assembled from many GWAS. And we found that NMD-QTLs are more likely to be disease-related.

2. I am concerned about rate of false calls of NMD QTLs. dNMD-QTLs are called when the association is only observable for NMD-targeted transcripts but not for non-NMD transcripts.

a. It is not clear what 'observable' means here? q-value < 0.05?

b. If so, then if q-value<0.05 is observed for NMD-Txs and q-value>=0.05 is observed for the non-NMD Txs, a dNMD-QTL is called? If this is the case, it is very possible to get such effect just due to difference in the power of the tests of the NMD and non-NMD Txs. To get some estimate of false calls, the authors could, as a control, repeat the same analysis on the non-NMD genes; split each such gene to two random sets of transcripts (in a way that simulates the division of transcripts of NMD genes to NMD and non-NMD transcripts), and compare the prevalence of "NMD" QTLs detected by such control to the one obtained on the NMD genes.

Responses: Thanks for the comments. We now added more details and more analysis about false positive control.

(a) Yes, q-value was used in FastQTL (Halit et al. (https://doi.org/10.1093/bioinformatics/btv722)) for false discovery control. Following the GTEx v7 settings, a gene-variant was declared a significant pair if the q-value ≤ 0.05. pNMD-QTL candidates were identified as gene-variant pairs showing both q-values ≤ 0.05 for the NMD- transcript group and the non-NMD transcript group. And then, we applied quantile regression to the original expression values without the inverse normal transform to double-check effect size directions and significances (quantile regression nominal p-value ≤ 0.05). dNMD-QTL candidates were identified as gene-variant pairs showing a q-value ≤ 0.05 in the NMD-transcript group, but a q-value > 0.80 in the non-NMD transcript group. Then, the quantile regression model was applied to the original expression to ensure a significant slope for NMD-transcripts (quantile regression nominal p-value ≤ 0.05) and a non-significant slope for non-NMD- transcripts (quantile regression nominal p-value > 0.95).

(b) As we explained in (a), the non-significance declaration requires q-values > 0.8 and quantile regression nominal p-value >0.95. We also added the control experiment mentioned by the reviewer: We randomly selected 6000 non-NMD genes. The transcript isoforms from these genes were randomly grouped into two groups to mimic the NMD-transcript and non-NMD- transcript groups. Then we performed the same NMD-QTLs calling procedures for this control setting. We tested the Adipose Subcutaneous tissue. Using the same criteria mentioned in (a), 2228 pNMD-QTLs were identified in the random control. That is about 12% of the total number of pNMD-QTLs discovered for Adipose Subcutaneous in our results (18,571). Thus, the actual FDR is about 0.12. For dNMD-QTLs, the estimated FDR based on the control set is about 0.17 for Adipose Subcutaneous.

3. The analysis on the overlap between splicing-QTLs and NMD-QTLs seems circular to me: the two tests are interlinked and are actually based on examination of the highly coupled features. This is exemplified by the NMD-genes with direct evidence for AS-NMD coupling. Consider such case, in which the gene has only one NMD transcript. The test for association with the level of that transcript is highly coupled with the test for association of the exon that defines that transcript (the exon containing the PTC). So statistically, these two highly related tests are bound to yield highly overlapping results.

Responses:

Thank you for your feedback. We fully agree that due to the nature of AS-NMD coupling, splicing-QTLs and NMD-QTLs are expected to be highly overlapped. The results we have presented here demonstrate that our identified NMD-QTLs are most likely to be true positives since they do exhibit a strong association with alternative splicing, as expected. In addition, our results reinforce the notion that AS-NMD coupling is pervasive in NMD regulation. We now added more discussion in the revision.

4. As for the enrichment of NMD-QTL for miR BSs compared to eQTLs:

a. As TragetScan miR BS are located in 3'UTRs, only 3'UTR SNPs should be included. If this is done, do NMD-QTLs still show enrichment for miR BSs compared to eQTL?

b. Figure 9: the label of the Y axis in panels a, c and e says 'enrichment'. But the Y axis doesn't show enrichment. In addition, a global test for enrichment was done, but not per tissue. Are all tissues show significant enrichment?

Responses:

We apologize for the confusion caused by the names of the Y axes in Figure 9. Here, we used the word "enrichment" in a general sense. To clarify, two types of statistics were calculated to evaluate the enrichment of NMD-QTLs with regard to miRNA and RBP binding sites. In figures 9(a), (c), and (e), to rank each miRNA/RBP, the Y-axis is the number of base-pair binding sites found to be NMD-QTLs divided by the total base-pair length of the binding sites for the considered miRNA/RBP. In figures 9(b), (d), (f) the Y-axis is defined as the number of NMD- QTLs/eQTLs discovered in the miRNA target or RBP binding sites, divided by the total number of NMD-QTLs/eQLTs discovered for a considered tissue. Also, in figures 9 (a) (c) (e), top miRNA/RBPs shared between dNMD-QTLs and pNMD-QTLs are orange colored.

(a) Thanks for the suggestions. We used only 3’UTR NMD-QTLs/eQLTs to calculate the percentages of them residing in miRNA targets. The same conclusions are observed: NMD- QTLs still show enrichment for miRNA binding sites compared to eQTLs (Supplementary Fig. 5).

(b). We changed the Y-axis descriptions of Figure 9 to make the two statistics discussed here clear. Regarding the tissue-wise significance, our analysis in figures 9 (b), (d) and (f) is at the tissue level. Each dot in the swarmplot represents the % of NMD-QTLs/eQLTs residing in the binding sites for a specific tissue. So tissues close to the bottom whiskers are less significant, and tissues higher above show more significant enrichment. While in figures 9(a) (c) (e) we pooled NMD-QTLs from all tissues into a nonredundant list to rank individual miRNAs/RBPs

5. For NMD-QTLs that were detected in many tissues (say, >20), could the authors examine and characterize the consistency of allele effect across the tissues?

Responses:

Thanks for the suggestion. A total of 2923 pNMD-QTLs were detected in more than 20 tissues. Remarkably, we found the allele effect to be highly consistent across tissues. For 62.9% (1840 out of 2923) of these pNMD-QTLs, the allele effect was 100% consistent across tissues, i.e., the allele effect sizes of a particular pNMD-QTL were all negative or all positive among the tissues it was detected. About 28.7% (840 out of 2923) of them were about fifty-fifty: the allele effect sizes were positive in ~50% of tissues and negative in the remaining ~50% of tissues. A total of 17,709 dNMD-QTLs were detected in more than 20 tissues. The allele effects were even more consistent across tissues. For 82.14% (8888+5659=14547 out of 17709) of these dNMD-QTLs, their allele effects were all consistent across tissues that they were discovered: 8888 of them were all negative, and 5659 of them were all positive.

6. In many places, the claims made by the authors should be toned down. For example, p11 line 43 "suggesting that NMD regulation is an essential component"; p12 line 48: ' .. is a key component of NMD regulation …'; p13 20: 'interplay closely with DNA repair and is essential in ensuring genome integrity'. Such strong claims should be based on some quantification of importance (or experimental validation). In the lack of such supporting evidence, the claims should be much weakened.

Responses:

Thanks for the comment. We toned down the statements that need validation to claim.

7. One limitation of the analysis performed in this study is the consideration of all gene's transcripts annotated as NMD-transcripts collectively. For an NMD-gene with multiple NMD-Txs, it is possible that an NMD-SNP would affect only one of the NMD-Txs, and therefore, its effect would be diluted when considering all NMD-TXs together. This limitation could be discussed in the Discussion.

Responses:

Thanks for the suggestion, we now added this to the discussion.

**2^nd^ round**

**Reviewer 1**

The authors have sufficiently addressed all my comments, I suggest publication of the manuscript in its current form.

**Reviewer 2**

The revised version is improved and has addressed many of the concerns I have raised.
- The demonstrated consistency in the direction of the allelic effects over tissues provides a good support.

- In such exploratory study, it is important to have some general assessment of false positive. Therefore, the added control (based on randomly selected non-NMD genes) is important.

- The authors opted not to perform a colocalization analysis with GWAS signals. Performing colocalization would indeed require much effort, but, in lack of experimental validation, could point strong candidate variants with causal effect on NMD.

- Fig 4a shows that NMD-QTLs are more likely to overlap disease-associated SNPs compared to ‘regular’ eQTLs. Can the authors examine if this enrichment stem from location differences? If both GWAS and NMD-QTLs tend to be located more closely to genes’ TSS than eQTLs, than the increased overlap could be simply explained by this trend. Can the authors (a) test if there are differences in distance distribution? And if there are (b) Does the enrichment hold also after controlling for it?

**Authors’ response**

Reviewer #1: The authors have sufficiently addressed all my comments, I suggest publication of the manuscript in its current form.

Responses: We thank you very much for your effort and time in reviewing our work. We appreciate your insightful comments and constructive feedback throughout the review process.

Reviewer #2: The revised version is improved and has addressed many of the concerns I have raised.

- The demonstrated consistency in the direction of the allelic effects over tissues provides a good support.

- In such exploratory study, it is important to have some general assessment of false positive. Therefore, the added control (based on randomly selected non-NMD genes) is important.

Responses: We thank you very much for dedicating your valuable time and effort to reviewing our work. We appreciate your insightful comments and constructive feedback throughout the review process.

- The authors opted not to perform a colocalization analysis with GWAS signals. Performing colocalization would indeed require much effort, but, in lack of experimental validation, could point strong candidate variants with causal effect on NMD.

Responses: Despite the challenges associated with implementing existing colocalization analysis tools, we have made significant progress and are pleased to present examples of colocalization between NMD-QTLs and GWAS signals using the ezQTL tool (https://analysistools.cancer.gov/ezqtl/#/home).

In one specific instance, we performed colocalization analysis between dNMD-QTLs derived from adipose (subcutaneous) tissue, targeting gene ENSG00000243696, and GWAS signals associated with body mass index (BMI) (Yengo et al., 2018). Our analysis highlighted SNP rs6769789 as a potential causal NMD-QTL for BMI, with a posterior probability of 0.846 using the HyPrColoc colocalization analysis (cutoff: 0.5), and a posterior probability of 0.023 using the eCAVIAR colocalization analysis (cutoff: 0.01).

In another example, we examined the colocalization between dNMD-QTLs identified in brain (cerebellum) tissue, targeting gene ENSG00000004534 (RBM6), and GWAS signals associated with the intelligence trait (Sniekers et al., 2017). We identified SNP rs11706370 as a candidate causal SNP with a posterior probability of 0.7576 using HyPrColoc. Furthermore, SNP rs2003485 surpassed the colocalization threshold in eCAVIAR, with a posterior probability of 0.0147.

These examples underscore the significance of NMD-QTLs in complex traits. These have been added to pages 14-15 with Supplementary Figures 3-4. To facilitate further exploration, we have provided ezQTL input files for our NMD-QTLs (https://github.com/QCBChenLab/NMD-QTL ), enabling users to investigate their own loci of interest and GWAS traits.

- Fig 4a shows that NMD-QTLs are more likely to overlap disease-associated SNPs compared to ‘regular’ eQTLs. Can the authors examine if this enrichment stem from location differences? If both GWAS and NMD-QTLs tend to be located more closely to genes’ TSS than eQTLs, than the increased overlap could be simply explained by this trend. Can the authors (a) test if there are differences in distance distribution? And if there are (b) Does the enrichment hold also after controlling for it?

Responses: Thank you for the suggestion. We conducted the recommended analysis. We observed a tendency for NMD-QTLs to be located in closer proximity to genes’ TSS compared to eQTLs, with a notable enrichment within the 500kbp region of the TSS (newly added Supplementary Figs. 2a-b). Considering that GWAS SNPs also tend to be located near the gene's TSS (Brodie et al. 2016), we investigated whether the enrichment of disease-associated NMD-QTLs could be attributed to their location preferences. To examine this, we categorized NMD-QTLs and eQTLs into three groups based on their distance from the TSS: <100k, 100k-500k, and 500k-1M. It was consistently observed that NMD-QTLs showed a higher likelihood of overlapping with disease SNPs (Supplementary Figs. 2c-e), although the enrichment signal for dNMD- QTLs was slightly weaker in the >500k group (Supplementary Fig. 2e). These have been added to Page 12.

**3^rd^ round**

**Reviewer 2**

The authors have addressed my additional comments.
As for the colocalization analysis, I understand the difficulties and limitations and recommend publication of the manuscript. A systematic colocalization analysis will be left for follow up studies (and the supplementary data that is now added allows doing that).

Yet, I do have a last comment with respect to the specific two examples for localization cases that were added to the ms. The example shown in Fig S3 is clear and convincing. However, the one shown in Fig S4 is nontrivial to me. Two distinct clusters of variants are apparent which may against the colocalization of the GWAS and dm-QTL signals at this locus. As this is the 2nd and last review cycle, there is no place for further clarification questions from my side. Therefore, I would suggest removing the example shown in Fig. S4 from the manuscript (unless the authors can fully explain the two clusters, and how this pattern supports colocalization).

**Authors’ response**

1.Regarding the concerns raised by Reviewer 2 about the two clusters in Fig. S4, we have carefully considered the comments. According to the ezQTL website, "For the matched alleles in both the QTL and GWAS datasets, we expect to observe a strong positive or negative correlation for a colocalized locus." The negative correlation depicted in Fig. S4 between our NMD-QTL z-scores and the intelligence GWAS z- scores suggests the presence of colocalization signals. Through formal tests, we identified SNP rs11706370 as a candidate causal SNP with a posterior probability of 0.7576 (HyPrColoc, 0.5 cutoff) and SNP rs2003485 with a posterior probability of 0.0147 (eCAVIAR, 0.01 cutoff). However, to exercise caution, we have decided to remove Fig. S4 as suggested by Reviewer 2. We have provided ezQTL input files for our NMD-QTLs, enabling users to investigate their own loci of interest and GWAS traits
